# Education Research: Teaching Neurologic Emergencies Through Serious Games: A Randomized Comparative Intervention Study

**DOI:** 10.1212/NE9.0000000000200217

**Published:** 2025-06-11

**Authors:** Maximilian Heidrich, Helena-Fee Gudorf, Kristoph Rauchstaedt, Andreas Breuer-Kaiser, Ralf Gold, Lars Tönges, Anne-Sophie Biesalski

**Affiliations:** 1 Department of Internal Medicine, Augusta Kliniken Hattingen, Bochum, Germany;; 2 Faculty of Medicine, Ruhr-University Bochum, St. Josef Hospital, Bochum, Germany;; 3 Department of Orthopaedics and Trauma Surgery, Knappschaftskrankenhaus Bochum-Langendreer, Ruhr University Bochum, Germany;; 4 Department of Anesthesiology, Ruhr University Bochum, St. Josef Hospital Bochum, Germany; and; 5 Department of Neurology, Ruhr-University Bochum, St. Josef Hospital, Germany.

## Abstract

**Background and Objectives:**

Serious games (SGs) are increasingly used in education, although data on their use in neurology education are limited. This study evaluates SG effect on knowledge retention, subjective impression of decision making, and learner satisfaction.

**Methods:**

Using a 6-step approach to curriculum development, we designed a digital interactive course as a SG, incorporating realistic video simulations to teach neurologic emergencies. A randomized intervention study compared the SG method (intervention) with clinical case seminars (seminar groups B and C) and no instruction (control group). Knowledge retention was assessed through multiple-choice (MC) tests immediately and 3 weeks postinstruction. Secondary measures included student satisfaction and usability. Descriptive statistical analyses were performed using IBM SPSS Statistics for Windows, Version 29.0, and free-text responses were analyzed qualitatively.

**Results:**

The survey initially included 77 students (control, n = 16; SG, n = 32; seminar control, n = 29), with 57 completing the follow-up survey. Scores on the MC test were similar immediately after the course (SG: 70.1%, Seminar Group B: 65.0%, Seminar Group C: 67.0%) and declined less for the SG (4.1%) than the seminar groups (10.9% for B, 5.5% for C). Likert scale responses exhibited higher satisfaction and usability in the SG group, with 93.5% of SG participants reporting a reduction in fear of clinical emergencies. Feedback from the SG participants was mostly positive, with many commenting on the engaging structure of the course.

**Discussion:**

Video-based SGs have shown efficacy in teaching neurologic emergency medicine. SG-acquired knowledge is more sustained than that acquired through traditional teaching formats and is well-received by Generation Z students.

## Introduction

A serious game (SG) is designed to convey knowledge in an engaging and interactive manner. One of the earliest forms of SGs was flight simulator training. SGs are gaining traction in medical education offering effective training in a safe yet realistic environment.^1^ They have been shown to be more effective than traditional methods for short-term learning in healthcare in some cases...^2^

SGs boost learners' self-confidence^3^ and enhance situational understanding, allowing for the training of clinical reasoning, traditionally taught through case studies and simulations.^4,5^ SGs also incorporate “stealth learning,” where enjoyment motivates learning,^6^ enhancing self-directed study. This approach allows skill acquisition without the risk of patient harm or exposure to judgment.

SGs are used in various medical fields,^7^ although there is limited focus on their role in medical education, particularly in neurology. Despite their potential, SGs must be used judiciously as tools in medical teaching.^8^

They are especially beneficial in subjects such as neurology,^9^ which is perceived as difficult to master.^10^ Integrating e-learning into traditional methods may improve neurology education.^11-13^ Interactive teaching can also address the shortage of young people entering neurology, which is growing rapidly in Germany,^14,15^ with an increasing demand for specialists to manage new therapies for a growing patient population.^16^

As neurologic emergencies become more prominent,^17^ education in this field is crucial. Digital methods can alleviate challenges in emergency training and reach a broader audience. We developed a digital SG course to improve instruction in neurologic emergencies at Ruhr-University Bochum (RUB), aiming to create a more lasting, effective learning experience.

## Methods

### Methodologic Background

To develop the course, we adhered to a **six-step approach to curriculum development,**^18^ encompassing **problem identification**, **needs assessment**, **goals and objectives**, **educational strategies**, **implementation,** and **evaluation.** Owing to the detailed, multistep nature of this process, this section summarizes the key decisions made in steps 1 through 4. The full description of steps 5 and 6 can be found below, with further details provided in the eMethods.

### Problem Identification

Initially, we identified the difficulties faced by students and young professionals in recognizing and treating acute neurologic emergencies in a clinical context as a central problem. Furthermore, during residency, training periods are brief and unstructured, requiring young professionals to acquire their skills through a “learning by doing” approach.^19^

Through our targeted **needs assessment,** we selected students nearing the end of their medical studies because of their extensive in-hospital experience and ability to integrate knowledge from other departments. These students face challenges when managing neurologic emergencies, making the end of their studies the perfect moment to combine theoretical knowledge with practical skills. Overall, the recently updated version of the National Competency-Based Catalog of Learning Objectives for Medicine (NKLM), published on April 27, 2021,^20^ and the proposed new medical licensing regulations^21^ have not been fully integrated at RUB. Moreover, the COVID-19 pandemic, among other factors, has prompted the digitalization of teaching at numerous universities.^22^

### Goals and Objectives

We aimed to train students to manage neurologic emergencies within a realistic digital clinical environment, including internal clinical processes, such as emergency imaging and intensive care unit transport. The course covered epidemiology, etiology, diagnostics, therapy, prognosis, and key red flags for each condition. Aligning with NKLM objectives, our approach focused on “epileptic seizure,” “acute stroke,” and “unclear unconsciousness” and developing case scenarios based on these conditions.

### Educational Strategies

Considering these objectives, we determined that an SG based on realistic video simulations would be the most effective approach.

### Case Scenarios and Video Scripts

**Figure 1:**
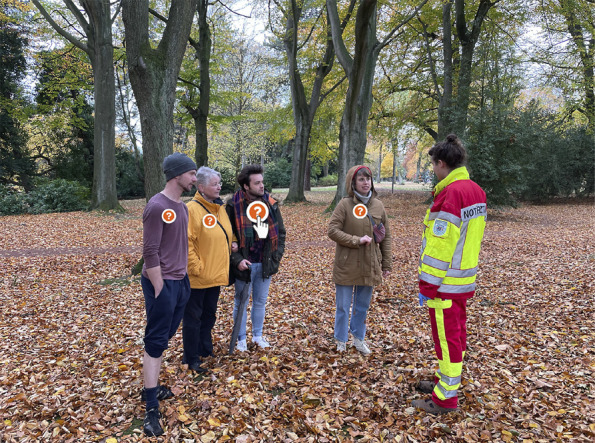
Interactive Survey of a Third-Party Anamnesis With Bystanders in the Serious Game

**Figure 2:**
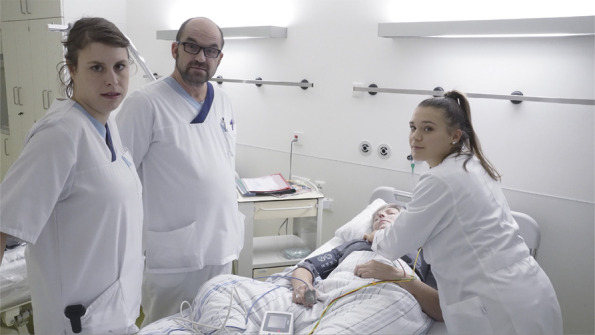
Introduction to Case Scenarios Begins With a Concise Overview of Preceding Events Each scenario unfolds progressively, interrupted by interactive elements or decision points. During these instances, protagonists address the player directly through the camera, soliciting input (e.g., a nurse inquiring, “What medication should I give now?”). This recording exemplifies such an interaction when the user is being directly addressed.

Each case scenario had specific learning objectives, depicting a “typical” progression of a neurologic emergency with integrated disease-specific complications. We planned to provide various reference and information sources for later questions (e.g., third-party anamnesis for an epileptic seizure; Figure 1) and included interruptions to prompt clinical decisions or alter the case progression (Figure 2). Using detailed characters, dialogues, and plots, we incorporated dramatic and humorous elements to engage students and enhance their enjoyment of the course.

### Shooting and Technical Process

Videos were produced in collaboration with a production company, using the facilities at the RUB simulation center (SimZ) at St. Josefs' Hospital in Bochum to create a realistic clinical environment. Clothing and props, including medical equipment, were provided by the simulation center or borrowed from the clinic. The roles of the patients and staff were portrayed by a combination of semiprofessional and professional actors.

Editing and dubbing were handled by the production company. On receiving the video files, we incorporated them into the “H5P” computer program, which is integrated into the RUB-Moodle-Platform.

The project was financed by the medical faculty at the RUB using funds for quality improvement (application no: 2021-006; object ID: 8008900477).

### From Video Cases to an SG: Additional Content and Interactive Components

**Figure 3:**
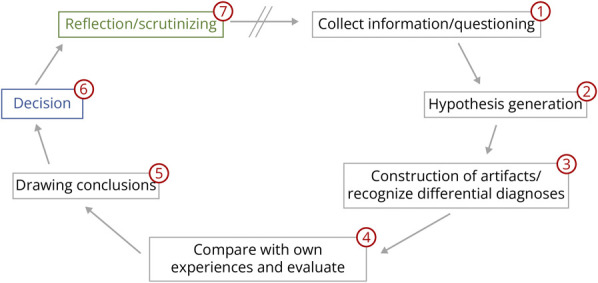
Structure of the Interactive Decision Pathway

**Figure 4:**
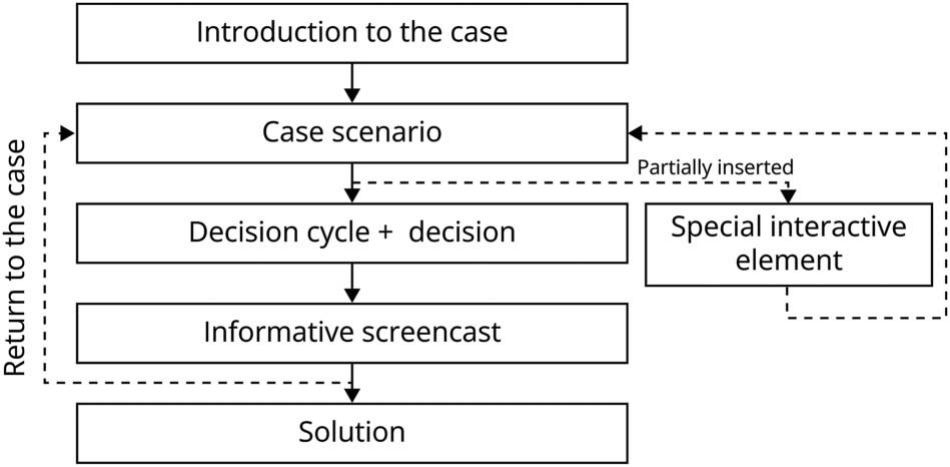
Schematic Representation of a Case Presented in the Serious Game

To provide a structured approach to clinical reasoning, the videos were paused at predetermined critical points. For instance, a scene might end with a nurse directly addressing the students after a key development, such as the patient experiencing another epileptic seizure, and asking which medication should be administered (Figure 2). This interruption was followed by an “interactive decision pathway” based on the hypothetico-deductive model of clinical reasoning^23^ designed to guide students in re-evaluating the case to make the necessary clinical decision (Figure 3). Students were encouraged to take written notes on their thoughts and conclusions as they systematically reviewed the case information. The final step of the decision pathway involved choosing the correct course of action, including the appropriate medication, dosage, and application method, from a list of options provided. After each decision, the steps were reviewed using an informative screencast video. These videos explored the intricacies of the situation, discussing potential pitfalls and weighing various options (e.g., choosing a diagnostic instrument). They also provided additional information on the topic, including insights from recent studies and relevant guidelines. After dealing with the initial question (e.g., which medication for epileptic seizures), the video seamlessly continues with the previous events (Video and Figure 4).

10.1212/200217_Video_1 Video Download Supplementary Video 1 via http://dx.doi.org/10.1212/200217_Video_1

### Implementation and Evaluation

To validate and assess the SG, we conducted a randomized intervention study comparing the effect of the SG on epileptic seizures and ischemic strokes with that of a traditional clinical case seminar. Data were initially collected through a multiple-choice (MC) test and a questionnaire distributed immediately after the educational intervention, followed by a follow-up survey 3 weeks after. Participants were assigned to 1 of 3 groups: (1) SG group, (2) clinical case seminar group (in subgroups of up to 15 students each), and (3) a control group (received no intervention).

### Standard Protocol Approvals, Registrations, and Patient Consents

The study was approved by the RUB Ethics Committee (application no: 21-7444, approved on March 3, 2022) and conducted in accordance with the ethical standards of the Declaration of Helsinki.

### Inclusion Criteria

Students in their practical year at RUB were invited to participate in the study. The practical year represents the sixth and final year of medical studies in Germany after the completion of the theoretical component and the second state examination, which includes a written test. During this year, students undertake three 4-month rotations in internal medicine, surgery, and a specialty of their choice, either in a hospital or a private practice. They are expected to assume medical responsibilities and manage patients under close medical supervision. Participants were recruited through the email distribution list of the university and internal advertisements within the clinic. At the time of recruitment, 205 students were in their practical year at RUB, all of whom had completed the same human medicine program, including the first and second state examinations. The study was conducted on the RUB campus, with follow-up conducted online. Participants were randomly assigned to one of the following groups using a lottery-based method: “Reference group,” “Serious game group,” “Seminar group B,” and “Seminar group C.” Students in the Reference group were involved in data collection without any preceding teaching or intervention. The SG group independently studied the material through the SG after a brief technical introduction. In the 2 seminar groups, instruction followed the “clinical case discussion” format, conducted in small groups with approximately 15 students and 1 lecturer. The PowerPoint presentation (PPT) used in the seminar groups contained the same information as presented in the SG but without displaying images of patients or clinical surroundings. Both lecturers received the PPT, were verbally trained beforehand, and did not access the SG material to avoid any unintentional bias.

After the conclusion of each course, data collection occurred in 2 phases: immediately after the course and 3 weeks later. The evaluation involved a test assessing factual knowledge on the topics of epileptic seizures and ischemic stroke, comprising 37 MC questions. The number of correct answers in the MC test served as a primary end point for the study (eTable 1). To validate the test procedure, a detailed item analysis was conducted to enhance the clarity, selectivity, and reliability. Subsequently, the questions were reviewed by several students and a neurology lecturer to identify any potential errors. In the course of the study, 11 of the original 37 MC questions were excluded, as they either lacked sufficient discriminatory power or were found to be ambiguously worded during the test. In addition, the clinical reasoning abilities of the participants were assessed using the Script Concordance Test.^24,25^ Third, we assessed SG usability, individual satisfaction with the respective teaching format, and individual learning behavior. The evaluation sheet incorporated a Likert scale, free-text entries, and specific questions on the teaching formats (Appendix). Additional inquiries about learning behavior and the participants' prior experiences aimed to establish the comparability of the intervention groups and identify potential confounding factors. To minimize bias, participants were prevented from communicating with each other between randomization and the conclusion of the initial data collection. The follow-up test aimed to explore and compare the sustained retention of knowledge acquired through the SG or the clinical case seminar. Both data collections were anonymized but could be cross-referenced using the individual codes.

### Statistical Analysis and Evaluation of Free-Text Responses

Statistical analysis was conducted using IBM SPSS Statistics (version 28). Descriptive statistics were first used to calculate the means, SDs, and frequencies. In addition, inferential statistical analysis was performed to examine the differences between groups. The differences in the MC test results between the intervention groups (SG and seminar groups B and C) and between the time points (t1, t2) were analyzed using *t* tests for independent and paired samples. A significance level of *p* < 0.05 was used to assess statistical significance.

The Likert scale results (eTable 2) were also evaluated descriptively. The categories “strongly agree” and “agree” were combined as agreement, whereas “slightly agree” and “disagree” were categorized as disagreement. These responses were then analyzed by group and time points to identify differences in satisfaction and perceived usefulness of the teaching methods.

For the evaluation of the qualitative data from the free-text responses, a simplified form of qualitative content analysis according to Mayring^26^ was applied. A deductive coding system was first developed, followed by an inductive expansion of the system to capture unexpected themes or patterns in the students' responses. Each response was coded according to these categories, and frequencies and key themes were identified and evaluated descriptively.

### Data Availability

Data that were not included in the article because of space constraints can be shared (anonymized) on request from any qualified investigator for the purpose of replicating procedures and results.

## Results

### Analysis of Group Characteristics and Previous Experience

In the initial data collection (t1), 77 students participated, of whom 16 were part of the Reference group and engaged solely in the initial survey. A total of 57 students participated in the follow-up survey (t2), whereas 4 (5.2%) dropped out. The participants were randomly assigned to the following groups: “Reference group” (n_t1_ = 16, n_t2_ = 0), “SG group” (n_t1_ = 32, n_t2_ = 31), “seminar group B” (n_t1_ = 15, n_t2_ = 13), and “seminar group C” (n_t1_ = 14, n_t2_ = 13). The average age of participants in the intervention groups was approximately 27 years (x̄ ≈ 26.70 years, SD ≈ 5.30). The biological sex of the participants was 57.9% (n = 33) female students and 42.1% (n = 24) male students. By the time of the second survey, almost all students (94.7%, n = 54) were in their practical year, while the remaining 3 were in the clinical part of their studies (5th-10th semester). Moreover, 22% (n = 7) of the students in the SG group, 13% (2/15) in seminar group B, and 21% (3/14) in seminar group C mentioned having prior training, such as medical professional training. Participants were asked about the subjects they wanted to or had completed during their elective third year of practical training (eTable 3). Participants were asked about the subject area they were considering for their specialist training (eTable 4).

On average, the students mentioned that 78.4% of their usual learning occurred via online learning platforms, 17.0% with books, 1.8% with podcasts, and 2.7% with other teaching materials. Further specified distribution of the teaching media can be found in eTable 5.

### Evaluation of the MC Results

All groups participated in the MC test. We considered 26 of 37 questions for the overall assessment. The Reference group scored an average of 41.3% correct answers (x̄ ≈ 10.7* correct answers out of 26 questions per group), the SG group achieved 70.1% correct answers (x̄ ≈ 18.2*), Seminar group B reached 65.0% correct answers (x̄ ≈ 16.9*), and Seminar group C scored 67.0% correct answers (x̄ ≈ 17.4*). Follow-up analysis showed a decrease in correct answers to 66.0% (x̄ ≈ 17.2*) in the SG group, 54.1% (x̄ ≈ 14.1*) in seminar group B and 61.5% (x̄ ≈ 16.0*) in seminar group C. The drop in correct answers between the first and second surveys was 4.1% in the SG, compared with 10.9% in seminar group B and 5.5% in seminar group C. To answer the primary question of our study, we used a 2-sided *t* test to compare the SG group with the 2 seminar groups B and C at both survey time points. The following findings were observed: In the SG group, no significant changes were detected over time (SG group t1 vs SG group t2: *p* = 0.199). However, a significant knowledge loss was identified in the combined seminar groups B and C (seminar groups B/C t1 vs seminar groups B/C t2: *p* = 0.014). When comparing the 2 groups (SG group vs seminar group B/C), no significant differences were found at the first survey time point, t1 (*p* = 0.142). At the second survey time point t2, however, the difference was significant (*p* = 0.023). Finally, when analyzing the knowledge loss between the 2 groups (SG group vs seminar group B/C) across the survey time points t1 and t2, no significant difference was observed (*p* = 0.147). Table presents an overview of all the results from the MC test at both survey times. The associated questions can be found in the attached questionnaire; the results are summarized in eTable 1.

**Table:** Results From the Multiple-Choice Test at Both Survey Times

	Reference group	Serious game group	Seminar group B	Seminar group C	Seminar groups (B + C)	Two-tailed *p* value SG-group vs seminar groups
First data collection (t1), (%)	41.30 (171/414)	70.07 (583/832)	65.00 (253/389)	67.00 (244/364)	66.00 (497/753)	0.142
Second data collection (t2), (%)		66.00 (532/806)	54.14 (183/338)	61.54 (208/364)	55.70 (391/702)	0.023
Two-tailed *p* value t1 vs t2 (loss of knowledge)		0.199	0.040	0.167	0.014	Differences between t1 and t2 SG-group vs Seminar groups (B + C): 0.147

Abbreviation: SG = Serious game.

The data display the percentage of correct answers, with the number of correct responses in brackets, compared with the total responses. The gray data represent the individual results of seminar groups B and C.

### Evaluation of the Questions in the Likert Scale Format

**Figure 5:**
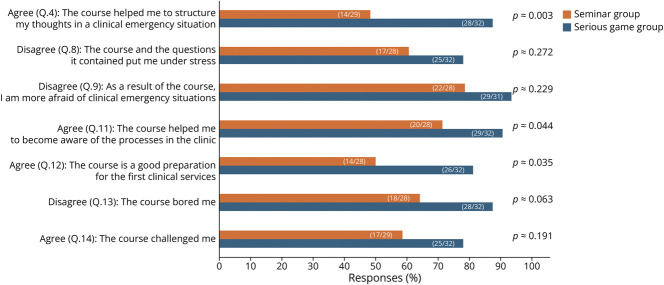
Selected Likert Scale Results From the First Survey (Full Results Are Available in eTable 2 and eFigures 1–15) * Numbers in parentheses represent: (the number of “Agree” or “Disagree” responses within the group/total responses for this question).

We presented 15 Likert scale questions, with options ranging from “totally agree “to” disagree.” For evaluation, responses of “totally agree” and “agree” were grouped as agreement, whereas those of “agree less” and “disagree” were categorized as disagreement. Figure 5 shows a selection of the most insightful Likert scale results. The full Likert scale data evaluation can be found in eTable 2 and eFigures 1–14.

To address the secondary objectives of this study, we explored subjective learner impressions of decision making, clinical reasoning, and confidence in emergency situations. The Serious game group was tested for significance against the combined results of the 2 seminar groups using a χ^2^ test.

On average, 42.6% (n = 26) of the participants disagreed with the statement that they had learned clinical decision making in emergency situations during their studies, while 36.1% (n = 22) agreed, and 21.3% (n = 13) provided a neutral response. In the Serious game group, 43.8% (n = 14/32) agreed, 18.8% (n = 6/32) were neutral, and 37.5% (n = 12/32) disagreed. In seminar group B, 33.3% (n = 5/15) agreed, 26.7% (n = 4/15) were neutral, and 40.0% (n = 6/15) disagreed. In seminar group C, 21.4% (n = 3/14) agreed, 21.4% (n = 3/14) were neutral, and 57.1% (n = 8/14) disagreed (*p* ≈ 0.4224).

A total of 87.5% (n = 28) of the SG group concurred that the course aided them in organizing their thoughts during a clinical emergency situation. By contrast, significantly fewer students from seminar groups B (46.7%; n = 7) and C (50.0%; n = 7) agreed with the same statement (*p* ≈ 0.0029).

Moreover, 62.5% (n = 20) of the students who participated in the SG affirmed that the course reduced their fear of clinical emergencies. Conversely, participants in the seminar groups showed different responses, with 42.9% (n = 6) in group B and 21.4% (n = 3) in group C agreeing with the statement (*p* ≈ 0.0591).

Similarly, when students were asked whether they became more fearful of clinical emergency situations after the respective course, most (93.5%; n = 29) SG participants disagreed with this statement (*p* ≈ 0.2289).

This study also aimed to illustrate the clinical workflows. After the SG, a substantial 90.6% (n = 29) of the students in this group agreed with the corresponding statement (“The course helped me to become aware of processes in the clinic”). In comparison, significantly fewer seminar participants (71.4%, n = 10) agreed with this statement (*p* ≈ 0.0440).

Moreover, a greater proportion of SG participants (81.3%; n = 26) felt that the course adequately prepared them for initial clinical practice compared with seminar group B (57.1%; n = 8) and group C (42.9%; n = 6) (*p* ≈ 0.0351).

The statement “The course bored me” was disagreed with by 87.5% of participants in the SG (n = 28), while 57.1% (n = 8) of participants from seminar group B and 71.4% (n = 10) from seminar group C also disagreed with this statement (*p* ≈ 0.0629).

Half of the SG participants (50.0%; n = 16) agreed with the statement “The course sparked my interest in neurology.” Meanwhile, 40.0% (n = 6) of the participants in seminar group B and 28.6% (n = 4) in group C agreed with the same statement (*p* ≈ 0.4242).

### Free-Text Analysis

The question “How did you like the course, do you have any comments or requests?” elicited 57 responses. Among the SG participants, 27 of 30 responses praised the course (for instance: “Super structured and interesting course!” or “It was fun and should also generally be included in student lessons”). Critiques about the time frame were expressed in 15 responses (10 times, e.g., “no realistic assessment of the processing time”) or technical issues (5 times, e.g., “audio volume is always automatically set to maximum”). Six SG participants expressed a desire for a hybrid event where the videos could be discussed interactively and questions asked (for example: “...I think that a kind of hybrid with a professor who is still present is even better”).

Among the seminar participants, 21 of 27 comments were positive, with feedback such as “...was very instructive.” or “...very interactive and nicely designed.” Conversely, 4 comments were related to the time frame indicating that it was “too long” or “...it was very exhausting for me to concentrate for so long after a practical year day.”

## Discussion

The SG described here represents a novel and distinctive teaching and learning format for neurology and neurologic emergency medicine in Germany. Both the didactic concept and technical implementation of the SG were reimagined and evaluated from the ground up, as there were no existing materials or prior experiences to draw upon. The conceptual approach based on the core cycle^18^ proved to be simple and applicable.

Despite facing various challenges, the creation and evaluation of the SG brought great satisfaction to everyone involved, and the intrinsic motivation of the teams remained high. The active involvement of students, emergency medicine instructors, and nursing staff significantly enriched this process. Aligning the content with the NKLM and adhering to the new licensing/approval regulations played a structuring role.

In retrospect, the SG emerged as a successful and innovative teaching initiative. However, certain elements of the course were identified as unnecessary or required didactic enhancement. The interactive decision pathway, intended to provide students with a structured approach to real clinical decisions, proved to be excessively time-consuming for repeated use within the SG. In addition, asking for the same basic information in subsequent decision cycles, even when the case history changed or new information was added, was deemed inefficient. Identifying and correcting individual errors in clinical reasoning of students' also presented challenges. Although the subsequent screencast addressed potential errors, personalized feedback could only be provided through artificial intelligence or direct interaction with a lecturer. Consequently, it would be more effective to incorporate the decision pathway into a seminar setting with direct instruction from a lecturer. Although the informative screencasts were well-received by students and deemed comprehensive, their creation was time-consuming, and the technical implementation (as a screen recording of a presentation) was suboptimal. A more effective approach would have been direct technical collaboration with the production company.

Therefore, a feasible long-term strategy could involve a hybrid model that combines video content with subsequent face-to-face discussions.

Previous research has highlighted several challenges in integrating SG into educational settings.^27-29^ Key issues include limited awareness among students and teachers, who may not be familiar with SG or may have biases against digital learning tools.^30^ Effective communication of the benefits of SG is needed to address these concerns.^29^ In addition, there is often a lack of technical knowledge required for developing and using SG.^31^ Providing proper training and promoting SG use in education can help overcome this barrier. Securing funding for the necessary infrastructure is another challenge. Strategic approaches to address these issues have been identified since 2010, including the following: (1) directly integrating developed SGs into the curriculum, (2) developing appropriate training programs, and (3) clarifying the potential benefits of SGs.^32^

During this study, we obtained insights into the usability of the SG at RUB. The age and sex distribution of the participants aligned with the overall student population at the university. The randomization process yielded mostly heterogeneous groups. Despite the small sample size, the survey remains representative.

The MC examination format used in this study is recognized for its validity and familiarity to students. Given their high validity and reliability, MC examination formats are commonly used in comparable medical didactic studies.^33^

Similar to previous studies, this study has a limitation.^34-36^ Owing to the relatively small number of participants, significant results were not observed. However, approximately one-third of the students in the relevant semesters participated, and the low dropout rate may be considered a positive factor.

All teaching formats showed a benefit compared with the group without intervention (*Reference group*). The SG group yielded the most favorable results, with a 70.1% success rate, compared with 65% and 67% in the seminar groups. Follow-up assessments further illustrated the sustainability of knowledge retention in the SG relative to that in seminar groups. These observations align with findings from previous studies where SG have proven to be more effective than traditional teaching formats.^37^

The Likert scale data supported these findings: students in the SG reported that the format helped them organize their thoughts during clinical emergencies and expressed higher satisfaction with the teaching method. This aligns with previous studies that have shown increased motivation, enjoyment, and satisfaction in game-based learning.^38^ A potential bias in our study is the elevated proportion of students in the SG group with an elective term in anesthesia (13/32 ≈ 40.6%), as these students may inherently have a greater interest in emergency medicine.

Participant feedback indicated that the seminars were exceptionally well-structured and engaging, with a strong focus on decision making. The lecturers showed high motivation and thorough preparation, which the participants found uncommon. Therefore, notable differences are expected when comparing these seminars to typical clinical seminars. Consequently, it is plausible that much more significant differences might be expected when comparing “typical” clinical seminars to the SG course.

One objective of the SG course was to train clinical decision making in a structured manner, an opportunity that many reported as lacking in their studies. The structured approach, along with the interactive decision pathway, helped reduce apprehension of students about making decisions in clinical emergency situations. However, based on the free-text analysis, among other considerations, the interactive decision pathway proved to be too time consuming for repeated use within the SG course and is better suited as an element under the guidance of a lecturer.

A distinctive aspect of the SG course, in contrast to the seminar group courses, was the video-based presentation of clinical processes (for example, registering a cranial computer tomography, communicating with the emergency services). The SG course provided participants with the chance to encounter a realistic clinical situation without apprehension and to sense the involvement in therapy decisions. This feature, along with the SG course overall, garnered positive feedback. The study results suggest that students would appreciate the inclusion of fundamental topics such as intraclinic procedures, economic and logistical considerations in everyday clinical practice, and interpersonal communication in the curriculum. We plan to incorporate these essential aspects of clinical patient care into our future neurologic teaching.

Overall, publications on SG in neurologic and emergency medicine teaching are scarce. Nevertheless, the overall advantages of SG as a teaching method are well-documented and are also apparent in our study.^2,3,37,38^ Some of our observations align with those of other groups: these include the enhanced sense of security among students resulting from simulation-based serious games^39^ as well as the increased sustainability of the knowledge they acquire.^40^

A key advantage of the SG course is its digital format, allowing accessible, self-directed learning from any computer with internet access, regardless of time or location.^41^

However, some students expressed in the free-text responses that they desired more individual feedback and direct support, elements not incorporated in our format. This implies that videos can be beneficially employed in face-to-face teaching. Moreover, combining video clips with personalized feedback is a promising method for acquiring practical skills.^42^

Developing the SG requires time, personnel, and financial resources. Nevertheless, the evaluation results indicate that this teaching format can effectively train decision making and impart clinical processes, with higher retention of acquired knowledge compared with traditional teaching formats, coupled with favorable acceptance by the Generation Z students.

A distinctive aspect of the course is the possibility of using individual components, such as realistic video sequences, in various contexts. This versatility allows for purely digital teaching methods, hybrid events (e.g., within a flipped classroom model), or integration into face-to-face instruction. The structure of the videos can also serve as a model for other departments to create their own courses.

We plan to continue this project at the RUB and within collaborative initiatives. Ongoing research in this field and the dedicated promotion of such projects are essential to establish digital (and hybrid) teaching methods in medical curricula.

## Glossary

CCT: cranial computer tomography
MC: multiple-choice
NKLM: National Competency-Based Catalog of Learning Objectives for Medicine
PPT: power point presentation
RUB: Ruhr-University Bochum
